# Predictive Factors Associated with Inappropriate Intravenous Proton Pump Inhibitors Use in Hospitalized Patients: A Case-Control Study

**DOI:** 10.3390/medicina61010010

**Published:** 2024-12-25

**Authors:** Niveen Khoury, David Stepensky, Naim Abu Freha, Mahmud Mahamid, Tawfik Khoury, Amir Mari

**Affiliations:** 1 Pharmacy Department, EMMS Nazareth Hospital, Nazareth 16100, Israel; nikhoury@gmail.com; 2 Department of Clinical Biochemistry and Pharmacology, Faculty of Health Sciences, Ben-Gurion University of the Negev, Beer Sheva 84105, Israel; davidst@bgu.ac.il; 3 The Institute of Gastroenterology and Hepatology, Soroka Medical Center, Faculty of Health Sciences, Ben-Gurion University of the Negev, Beer Sheva 84105, Israel; abufreha@yahoo.de; 4 Gastroenterology Department, Shaare Zedek Medical Center, Jerusalem 9103102, Israel; mmahamid@szmc.org.il; 5 Gastroenterology Department, Nazareth Hospital, Nazareth 16100, Israel; tawfikkhoury1@hotmail.com; 6 Gastroenterology Department, Galilee Medical Center, Nahariya 22000, Israel; 7 The Azrieli Faculty of Medicine, Bar-Ilan University, Ramat Gan 5211401, Israel

**Keywords:** intravenous, proton pump inhibitors, inappropriate prescription, clinical outcomes, hospital

## Abstract

*Background and Objectives*: Proton Pump Inhibitors (PPIs) are the most effective agents for treating acid-related gastrointestinal disorders. The prescription of an intravenous (IV) formulation of PPIs has increased dramatically. The aims of this study were to assess the appropriateness of IV PPI use and to define the risk factors and outcomes associated with its inappropriate use. *Materials and Methods*: A case-control retrospective study included all the hospitalized patients who received IV PPIs was conducted. Patient health records were reviewed, data were collected covering the period of the individual patients’ admission to the hospital until discharge or death, and over the 3-month post-discharge period. The appropriateness of the IV PPI use and the resulting clinical outcomes were analyzed. *Results*: Overall, 540 patients were analyzed. Among them, 130/540 (24%) had inappropriate PPI use in terms of indication, dosage, and duration of treatment vs. 410 patients who had appropriate indications. Two parameters were associated with inappropriate use: congestive heart failure (OR 1.77; *p* = 0.02) and prescription of IV PPIs by surgeons vs. internists (OR 1.53; *p* = 0.05). *Conclusions*: Inappropriate IV PPI use is still common in daily clinical practice. Significant predictors of inappropriate use were the presence of congestive heart failure, elderly age, current use of anticoagulants and antithromotics, and the cases managed by surgeons, naturally due to suspected upper gastrointestinal bleedings.

## 1. Introduction

Proton pump inhibitors (PPIs) have been proven to be an effective therapeutic option in a variety of upper gastrointestinal tract (UGI) disorders. Their introduction into clinical practice about thirty years ago has greatly improved the therapeutic approach to acid-related diseases for their well-recognized efficacy and safety [1]. Prescribed in an adequate intravenous (IV) dose, PPIs can maintain intragastric pH at 6 or above, so that peptic activity is minimized, and fibrinolysis is inhibited [2,3]. For most patients who require PPIs, oral treatment is effective to treat dyspepsia, mucosal ulcerations, reflux related symptoms, and complications as well as for the treatment of Helicobacter pylori infection. However, there are cases where hospitalized patients may require IV PPIs. The indications for IV PPIs are: treatment of perforated gastric/duodenal ulcers, peptic ulcer disease, grade III/IV esophagitis, stress ulcer prophylaxis in patients that cannot consume oral drugs or are critically ill and for bleeding ulcers [4]. The International Consensus Group and The European Society of Gastrointestinal Endoscopy (ESGE) Cascade Guideline recommends using high dose IV PPI following endoscopic hemostasis in patients with non-variceal upper gastrointestinal bleeding (NVUGIB), as this treatment has been demonstrated to reduce rebleeding from high-risk peptic ulcers. Also, high-dose pre-endoscopic IV PPI is recommended in patients presenting with acute UGIB awaiting upper endoscopy [5,6,7,8].

Since their introduction into clinical practice, despite the limited number of their well-defined indications, the use of PPIs continues to grow every year. This class of antisecretory therapy falls only behind the statins in total cost expenditure worldwide, estimated at over USD 11 billion annually [9]. The strong evidence supporting PPIs’ efficacy and their favorable safety profile may have dramatically contributed to over-prescription. This extensive growth poses serious queries about the appropriate prescription of these drugs worldwide and has created important problems for many regulatory authorities for two relevant factors: the progressive increase of the costs of therapy and the increased risk of drug-induced toxicity in the patients. Several studies were conducted in hospitals in the United States, Europe, and the Middle East to examine the use of IV PPIs, and to determine factors predicting inappropriate prescribing practice. These studies have demonstrated that 25–75% of patients receiving IV PPIs had no appropriate indication [4,7,10,11,12,13,14].

There is inadequate literature available about the prescribing patterns of IV PPIs in hospitalized patients. Identifying this unmet need, and in response to this gap of knowledge we conducted a retrospective study to assess the appropriateness of the IV PPI prescriptions patterns among hospitalized patients in a university hospital. We aimed to define and evaluate the risk factors associated with inappropriate prescription of IV PPIs related to the patients and to the prescribing doctors, as well as to evaluate patient-related clinical outcomes and predictive factors associated with inappropriate use of IV PPI.

## 2. Materials and Method

A case-control retrospective study was conducted at the EMMS (Edinburgh Medical Missionary Society) Nazareth hospital. The EMMS Nazareth hospital is a regional teaching hospital which is affiliated with the Faculty of Medicine, Bar-Ilan University, Israel. This study included inpatients who were prescribed IV PPIs during hospitalization between the years 2015 and 2019, according to the data in the electronic health records. The IV PPIs that were prescribed at the relevant period in the hospital were Pantoprazole and Esomeprazole.

We reviewed the electronic health records of 540 patients who met the following inclusion criteria: patients who were treated with IV PPIs during hospitalization in one of the hospital departments. We excluded from the study the patients who were younger than 18 years, pregnant women, oncologic patients, patients with liver cirrhosis, and patients with increased risk of bleeding (e.g., hemophilia, thrombocytopenia, and coagulation factors deficiencies).

The review process of the files has been performed by a clinical pharmacologist and validated in a separate review process by a senior gastroenterologist.

Patient health records were reviewed, and the data were collected covering the period of the individual patients’ admission to the hospital, until discharge or death. The extracted data included demographics, admission diagnosis, previous medical history, laboratory findings (kidney function, liver function, complete blood count, coagulation functions, and electrolytes). Moreover, PPI indication, dosage, and treatment duration, as well as the endoscopic or surgical findings, characteristics of the prescribing physician (specialty, status), other medications prescribed during the hospitalization (specifically: antiplatelets, anticoagulants and steroids) were all collected (Table 1). Furthermore, we analyzed the patients’ medical records from a national health database for three months after the discharge to observe the patient’s clinical outcomes. We checked the electronic health records for hospital readmission, emergency room visits, out-patient clinic visits, as well as family physician visits, and laboratory tests.

We compared the results between the two groups: patients who were prescribed IV PPIs appropriately or inappropriately, based on the definition of appropriate IV PPI use in Table 1 [10,11,12,13,14,15].

The following clinical outcomes were assessed and reported: gastrointestinal rebleeding rate, rehospitalization rate, diarrhea incidence, *Clostridium difficile* infection (CDI), pneumonia, renal impairment, and all-cause mortality.

Patients were classified based on their clinical presentation, endoscopic findings, and the reason for IV PPI use (UGIB and NUGIB).

The study protocol was approved by the Institutional Review Board (IRB) of the EMMS Nazareth Hospital (approval no. NZH012020).

### Statistical Analysis

The statistical analysis was performed using SAS Software, Version 9.4. Continuous variables were presented as mean ± SD, categorical variables were presented as counts and percents of the total (N, %). Logistic regression was used to calculate the univariate and multivariate odds ratios (OR). Firth’s methodology was used to deal with sparseness of data in some of the categorical predictors. Backward elimination was used to obtain the final model in the multivariate analysis. Two-sided statistical tests were used, and a *p* value less than 0.05 was statistically significant. All analyses were performed by an experienced statistician.

## 3. Results

### 3.1. Demographics and Baseline Characteristics

Overall, 540 patients were included in the study. Among them, 130 patients had inappropriate indication for PPI use (group A), as compared to the 410 patients who had appropriate indications (group B). In total, 24% of all IV PPI prescriptions given in the EMMS Nazareth hospital at the studied period had inappropriate indications, dosing, or duration of therapy. The average age in group A was 65.5 ± 19.4 vs. 52.7 ± 21.9 years in group B. The gender balance in groups A and B was similar (43.8% vs. 38.3% males, respectively). The rate of previous PPI use, chronic anticoagulation therapy, and antiplatelets use were higher in group A, as compared to in group B (see Table 2).

### 3.2. Clinical Characteristics and Laboratory Findings

**Figure 1:**
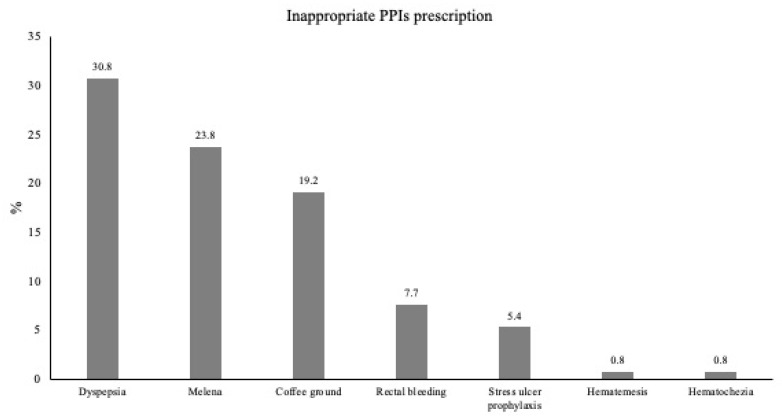
The incidence of inappropriate use of IV PPIs in specific clinical indications.

The most common cause for in-hospital admission was upper gastrointestinal symptoms (41.5% in group A, vs. 24.9% in group B). Similarly, the most common indication for PPIs prescription was UGIB (40.8% and 35.6% in groups A and B, respectively) (see Figure 1). Notably, 51.5% of the inappropriate prescriptions were prescribed by surgeons compared to 48.5% prescribed by the internist. A total of 92.3% of the inappropriate prescriptions were prescribed by junior doctors. The mean duration of treatment was longer in group A, as compared to group B (3.6 ± 1.6 vs. 3.0 ± 1.2, respectively). More IV PPIs were switched to oral during hospitalization in patients who had an inappropriate prescription (35.4% in group A vs. 24.6% in group B). Table 3 describes the clinical characteristics and the laboratory findings at the admission.

### 3.3. Univariate and Multivariate Analysis of Parameters Associated with Inappropriate Use

Based on a univariate analysis (see Table 4), several parameters were associated with inappropriate PPI use, including: age (OR 1.03, 95% CI 1.02–1.04, *p* < 0.0001), comorbidities such as diabetes mellitus (OR 1.68, *p* = 0.011), hyperlipidemia (OR 3.06, *p* < 0.0001), hypertension (OR 2.48, *p* < 0.0001), ischemic heart disease (OR 1.86, *p* = 0.006), congestive heart failure (OR 3.11, *p* < 0.0001), atrial fibrillation (OR 3.06, *p* < 0.0001), chronic renal failure (OR 1.96, *p* = 0.005), obstructive lung disease (OR 2.95, *p* = 0.0002), previous PPI therapy (OR 1.61, *p* = 0.04), chronic anticoagulation therapy (OR 2.3, *p* = 0.002), and chronic antiplatelet therapy (OR 2.52, *p* < 0.0001). There was no effect of steroids (*p* = 0.95) or non-steroidal anti-inflammatory drug (NSAIDs) use (*p* = 0.59) on the rate of inappropriate use of IV PPIs. Moreover, stress ulcer prophylaxis as an indication for PPIs was associated with a lower likelihood for inappropriate use (OR 0.08, *p* < 0.001).

Based on logistic regression multivariate analysis (see Table 4), two parameters were associated with inappropriate IV PPI use, including congestive heart failure (OR 1.77, 95% CI 1.07–2.93, *p* = 0.02) and the prescriber: surgeons vs. internists (OR 1.53, 95% CI 0.98–2.38, *p* = 0.05). The association between the stress ulcer prophylaxis as an indication for PPI use and the lower likelihood for inappropriate use was preserved (OR 0.07, 95% CI 0.03–0.16, *p* < 0.0001).

### 3.4. Three Months’ Follow-Up Results

Table 5 demonstrates the clinical outcomes of the three-month follow-up period. There was no effect on the rate of CDI, pneumonia, acute kidney injury, and diarrhea (*p* = 0.17, *p* = 0.24, *p* = 0.55, and *p* = 0.37), respectively. Moreover, there was no effect on recurrent hospitalization or recurrent bleeding.

## 4. Discussion

This retrospective case-control study showed that 24% of all IV PPI prescriptions had inappropriate use in a single hospital by one of the following means: indications, dosing, or duration of therapy.

The prevalence of inappropriate prescribing of intravenous PPIs in our study is substantially lower than reported in previous studies conducted in several hospitals worldwide [16,17]. The possible explanation for this could be due to the differences in the definition of the appropriate indications and the appropriate dosing in different studies [18]. We considered pre-endoscopic IV PPIs treatment in patients with suspected UGIB to be appropriate, which is similar to some previous studies [10,11,12,15], while another study considered this treatment inappropriate [7]. We also defined IV PPI use in suspected UGIB to be appropriate when it was discontinued within 72 h if no bleeding source was detected and UGIB was no longer suspected. The reason for this definition was to provide the doctors some flexibility, especially in the emergency room, and in case of difficulty in performing endoscopy due to patient’s acute physical condition. This definition is similar to Lai et al. [4], while other studies considered this prescribing to be appropriate if the infusion was stopped within shorter duration if no bleeding was confirmed [10,12], and other researchers did not consider this use to be appropriate [7].

In terms of dosage, the dose of 80 mg bolus of IV PPIs followed by 8 mg/h for 3 days from the time of endoscopy or onset of bleeding is considered appropriate in patients with UGIB. In addition, we considered the dose of 80 mg bolus followed by 40 mg twice daily infusion to be appropriate dosage, this is in accordance with other studies [19].

Another possible explanation for lower rate of inappropriate IV PPI use in this study, as compared to other studies, is the presence of an active gastroenterology department in our hospital (which probably was not available in other studies as it was not stated explicitly in the previous studies). This is reflected in the existence of on-call gastroenterologists, who are available for counseling and performing urgent procedures twenty-four hours a day, as well as the existence of treatment guidelines for treating active upper GI bleeding and stress ulcer prophylaxis in critically ill patients and bariatric patients. Finally, our pharmacists play an important role in implementing appropriate prescribing habits. They examine the medical records for most of the patients admitted to the hospital and evaluate the appropriateness of the indications as well as the dosage and the duration of treatment.

The mean duration of therapy in our study in the appropriate indication group was similar to that in a previous study which reported a median duration of therapy of 3 days for both stress ulcer prophylaxis and presumed UGIB [20]. However, a longer duration of therapy of 5–6 days were reported by others [11,12]. The possible explanation for the differences in the mean duration of treatment could be attributed to the differences in PPI indications in each study. In our study, the most common indication for IV PPIs was acute UGIB (40.8% and 35.6% in groups A and B, respectively). Moreover, we noted that stress ulcer prophylaxis as an indication for PPIs was associated with a lower likelihood for inappropriate use (OR 0.08, *p* < 0.001). This result is like the outcomes of a recent study [21]. Importantly, a quality measure by our hospital pharmacy is to daily assess the need of continuing IV PPI before issuing the medication.

When assessing the potential predictors for inappropriate IV PPIs prescribing, we observed that older age is a risk factor (OR 1.03, *p* < 0.0001). These results are in accordance with a recent study that reported older age as a predictor for inappropriate IV PPIs therapy [22]. This is probably because older patients are treated more aggressively and inappropriately, at least regarding treatment with PPIs, as they tend to have more co-morbidities and suffer from a clinically fragile condition.

Furthermore, we found that chronic anticoagulation and antiplatelet therapy was related to the inappropriate prescribing of IV PPIs [23]. These results differ from those of other studies that found that patients on chronic anticoagulation therapy were more likely to have IV PPIs prescribed appropriately [11]. Further studies are needed to explore the reasons for this controversy. Still, in clinical practice, the patient’s age, medical background, and chronic medications are all factors that support our attitude and approach. Assessing a patient in the emergency department with older age, complicated medical conditions, and polypharmacy is usually a very active approach with maximal activity by means of investigations and management by clinicians.

IV PPI was more inappropriately prescribed by surgeons. We assume this is due to the fact that surgeons generally treat cases of suspected UGIB, suspected perforated ulcers, and other emergency conditions. Most cases are generally managed by senior and junior doctors, however, generally the medication prescribing is done by junior doctors and trainees [7,10].

Moreover, background medical conditions have been linked to the inappropriate prescribing of IVPPI. Congestive heart failure was found to be associated with inappropriate IV PPI use, however we could not find any previous data like our findings, making our data important for clinicians.

Additionally, at three months follow up, inappropriate PPIs had no effect on the development of CDI, pneumonia, the worsening of kidney function, recurrent hospitalization, and recurrent bleeding. This might be addressed by the excellent safety profiles of PPIs, however, missing data are a potential issue in this case.

Putting all this together, this retrospective case-control study confirms that there is an inappropriate prescribing of IV PPIs in hospital practice. Although our findings show a lower prevalence of inappropriate prescribing than other studies, we should try to target our intervention towards staff education and the implementation of treatment guidelines among doctors of all specialties, especially surgeons.

Our study has some specific limitations. First, this is a retrospective study that relies on information in the electronic medical records, which may be incomplete. Second, though our data for each patient were as objective and complete as possible, a medical records review was performed by a single investigator, which can potentially introduce subjective bias. The follow up after discharge relied on electronic files on medical systems that may be incomplete due to reporting bias. Third, the definition of appropriateness of IV PPI used in our study differs in some respects from that in other studies and publications. Our definitions were derived from the guidelines and international consensus groups, considering the data in other studies conducted worldwide.

## 5. Conclusions

In conclusion, inappropriate IV PPI usage is still common in daily clinical practice, and it is more frequent in patients with UGIB as well as in elderly patients with cardi-ac conditions and current anticoagulant use. It is important to implement and enforce treatment guidelines and staff education in daily clinical practice to optimize drug uti-lization and appropriateness, as the inappropriate prescribing of IV PPIs in hospitals can extend to their inappropriate prescription in ambulatory settings. More prospec-tive multicenter studies are warranted to investigate the effect of inappropriate IV PPI use on morbidity and mortality, as well as its impact on health systems.

## Figures and Tables

**Table 1:** Definitions of the appropriate IV PPIs prescription.

IV PPI IN UGIB Appropriate indications for IV PPIs in UGIB UGIB that was diagnosed by endoscopy or surgery; Hemodynamic instability with evident clinical bleeding; Esophagitis grade C/D according to LOS ANGELES classification; Suspected UGIB treated with IV PPIs, but the treatment was stopped in less than 72 h because no bleeding source was detected; Appropriate doses of IV PPIs in UGIB Loading dose of 80 mg followed by continuous infusion of 8 mg/h for 72 h; Loading dose of 80 mg followed by 40 mg every 12 h. Duration of PPIs treatment in UGIB Treatment must be initiated as soon as possible and continued for up to 72 h after endoscopy for patients with high-risk stigmata of recent bleeding (e.g., active bleeding, visible vessel, or adherent clot). Patients without high-risk stigmata of recent bleeding can be switched to an oral PPI immediately after endoscopy.
IV PPIs in NUGIB Appropriate indications for IV PPIs in NUGIB Treatment for stress ulcer prophylaxis in mechanically ventilated patients or critically ill patients at the intensive care unit; Patients who are nil by mouth and need PPI treatment specially patients after bariatric surgery. Appropriate doses of IV PPIs in NUGIB 40 mg once daily. Duration of PPIs treatment in NUGIB As long as the patients are unable to take oral medications

**Table 2:** Demographics and baseline characteristics of the patients included in this study.

Parameter	Inappropriate IV PPI Use	Appropriate IV PPI Use	*p* Value
Number of patients	130	410	-
Age, mean ± SD	65.5 ± 19.4	52.7 ± 21.9	<0.0001
Male Gender, N (%)	57 (43.8)	157 (38.3)	0.26
Ethnicity: Arab, N (%)	121 (93.08)	380 (92.68)	0.985
BMI, mean ± SD	31.2 ± 6.3	38.9 ± 7.1	<0.0001
Medical history, N (%)			
Diabetes mellitus	57 (43.8)	130 (31.7)	0.01
Hyperlipidemia	8565.4)	156 (38.1)	<0.001
Hypertension	88 (67.7)	187 (45.6)	<0.001
Ischemic heart disease	39 (30)	77 (18.8)	0.01
Congestive heart failure	38 (29.2)	48 (11.7)	<0.001
Cerebrovascular disease	16 (12.3)	43 (10.5)	0.6
Atrial fibrillation	35 (26.9)	44 (10.7)	<0.001
Inflammatory bowel disease	1 (0.8)	4 (1)	1
Chronic kidney disease	35 (26.9)	65 (15.8)	0.006
Previous gastrointestinal bleeding	9 (6.9)	23 (5.6)	0.6
Peptic ulcer disease	7 (5.4)	19 (4.6)	0.8
Obstructive lung disease	26 (20)	32 (7.8)	<0.001
On PPI therapy, N (%)	36 (27.7)	79 (19.3)	0.049
Anti coagulant therapy before admission, N (%)	27 (20.8)	42 (10.2)	0.004
Antiplatelets therapy before admission, N (%)	60 (46.1)	104 (25.4)	<0.001
NSAIDS therapy before admission, N (%)	1 (0.8)	2 (0.5)	0.56
Steroids therapy before admission, N (%)	2 (1.5)	7 (1.7)	1

**Table 3:** Clinical characteristics and laboratory findings of the patients included in this study.

Parameter	Inappropriate IV PPI Use, N%	Appropriate IV PPI Use, N%	*p* Value
Cause of admission, N (%)			
Upper gastrointestinal bleeding	19 (14.6)	64 (15.6)	0.89
Bariatric surgery	0	169 (41.2)	<0.001
Lower gastrointestinal bleeding	7 (5.4)	6 (1.5)	0.02
Upper gastrointestinal symptoms	54 (41.5)	102 (24.9)	<0.001
Surgical emergency	9 (6.9)	11 (2.7)	0.03
Others	41 (31.5)	58 (14.1)	<0.001
Indication of PPI, N (%)			
Melena	31 (23.8)	69 (16.8)	0.09
Coffee ground	25 (19.2)	69 (16.8)	0.51
Hematemesis	1 (0.8)	16 (3.9)	0.08
Hematochezia	1 (0.8)	2 (0.5)	0.56
Rectal bleeding	10 (7.7)	15 (3.7)	0.08
Stress ulcer prophylaxis	7 (5.4)	177 (43.2)	<0.001
Dyspepsia	40 (30.8)	38 (9.3)	<0.001
Hemodynamic status, N (%)			
Stable	103 (79.2)	362 (88.3)	0.01
Unstable	27 (20.8)	48 (11.7)	
Physician who prescribed PPI, N (%)			
Internist	63 (48.5)	153 (37.3)	0.03
Surgeon	67 (51.5)	256 (62.4)	
Day of IV PPI prescription			
Day 1	88 (70.7)	323 (70.2)	
Day 2	4(18.3)	67(18.4)	0.2
Day 3	18(12.0)	42 (11.4)	
Physician experience, N (%)			
Resident	120 (92.3)	359 (87.6)	0.15
Senior	10 (7.7)	51 (12.4)	
Hemoglobin g/dL, mean ± SD	11.1 ± 3	11.8 ± 2.6	0.01
Creatinine mg/dL, mean ± SD	1.5 ± 1.3	1.2 ± 1.4	0.08
INR, mean ± SD	1.3 ± 0.8	1.2 ± 1.2	0.62
Platelets (× 10^9^), mean ± SD	278 ± 133	290 ± 115	0.37
PPI given on hospital, N (%)			
IV Pantoprazole	51 (39.2)	169 (41.2)	0.76
IV Esomeprazole	79 (60.8)	241 (58.8)	
Route of administration, N (%)			
Continuous	6 (4.6)	45 (11)	0.04
Non-continuous	124 (95.4)	365 (89)	
Number of days of PPI given, mean ± SD (range)	3.6 ± 1.6 (1–12)	3 ± 1.2 (1–10)	0.001
PPI switched to oral in hospital, N (%)	46 (35.4)	101 (24.6)	0.02

**Table 4:** Univariate and multivariate analysis of parameters associated with inappropriate use of IV PPIs.

	Univariate Analysis		
Parameter	Odds Ratio	95% CI	*p* Value
Age	1.03	1.02–1.04	<0.0001
Male gender	1.26	0.84–1.88	0.26
Body mass index	0.88	0.84–0.93	<0.0001
Diabetes mellitus	1.68	1.12–2.52	0.011
Hyperlipidemia	3.06	2.02–4.61	<0.0001
Hypertension	2.48	1.64–3.76	<0.0001
Ischemic heart disease	1.86	1.19–2.91	0.006
Congestive heart failure	3.11	1.92–5.04	<0.0001
Cerebrovascular disease	1.22	0.66–2.24	0.53
Atrial fibrillation	3.06	1.86–5.04	<0.0001
Inflammatory bowel disease	1.05	0.14–7.99	0.96
Chronic renal failure	1.96	1.23–3.13	0.005
History of GI bleeding	1.29	0.58–2.84	0.53
History of peptic ulcer	1.22	0.50–2.94	0.66
Obstructive lung disease	2.95	1.68–5.18	0.0002
Previous PPI therapy	1.61	1.02–2.54	0.04
Chronic anticoagulant therapy	2.30	1.36–3.91	0.002
Chronic antiplatelets therapy	2.52	1.67–3.79	<0.0001
Chronic NSAIDs therapy	1.89	0.18–19.74	0.59
Chronic steroids therapy	1.05	0.23–4.79	0.95
Hemoglobin at admission	1.11	0.84–0.97	0.005
Creatinine at admission	1.12	0.99–1.28	0.08
INR at admission	1.06	0.87–1.29	0.58
Platelets at admission	1	0.99–1	0.38
Hemodynamic status: unstable	1.99	1.18–3.34	0.009
Physician experience: senior	0.61	0.30–1.22	0.16
Physician who prescribed PPI: surgeon	1.56	0.43–0.95	0.03
Indication for PPI: melena	1.56	0.96–2.51	0.07
Indication for PPI: coffee ground	1.19	0.72–1.97	0.51
Indication for PPI: hematemesis	0.28	0.05–1.56	0.15
Indication for PPI: hematochezia	1.89	0.18–19.74	0.59
Indication for PPI: Stress ulcer prophylaxis	0.08	0.04–0.17	<0.0001
	Multivariate logistic regression analysis		
CHF	1.77	1.07–2.93	0.02
Stress ulcer prophylaxis	0.07	0.03–0.16	<0.0001
Physician’s specialty surgeon vs. Internist	1.53	0.98–2.38	0.05

**Table 5:** Results of the three months follow-up period.

Parameter	Appropriate IV PPI Use, N (%)	Inappropriate IV PPI Use, N (%)	OR	95% CI	*p* Value
Recurrent hospitalization	122 (32.2)	44 (37.9)	1.29	0.84–1.98	0.26
Recurrent bleeding	26 (6.7)	9 (7.3)	1.1	0.5–2.39	0.84
Diarrhea	33 (8.5)	14 (11.3)	1.37	0.71–2.66	0.37
Acute kidney Injury	52 (12.8)	19 (14.8)	1.18	0.67–2.1	0.55
Pneumonia	38 (9.74)	17 (3.71)	1.47	0.8 −2.7	0.24
Clostridium difficile infection	7 (1.8)	5 (4.03)	2.29	0.71–7.33	0.17

## Data Availability

Data available upon request from corresponding author.
